# West Nile Virus Surveillance, Guadeloupe, 2003–2004

**DOI:** 10.3201/eid1107.050105

**Published:** 2005-07

**Authors:** Thierry Lefrançois, Bradley J. Blitvich, Jennifer Pradel, Sophie Molia, Nathalie Vachiéry, Guillaume Pallavicini, Nicole L. Marlenee, Stéphan Zientara, Martial Petitclerc, Dominique Martinez

**Affiliations:** * Centre de Coopération Internationale en Recherche Agronomique pour le Développement, Prise d'Eau, Guadeloupe, French West Indies;; † Colorado State University, Fort Collins, Colorado, USA;; ‡ Agence Française de Sécurité Sanitaire des Aliments, Paris, France;; § Direction des Services Vétérinaires de Guadeloupe, Basse-Terre, Guadeloupe, French West Indies

**Keywords:** West Nile Virus, public health, flavivirus, equine, avian, guadeloupe, ELISA, epidemiology, caribbean

## Abstract

We conducted extensive surveillance for West Nile virus infection in equines and chickens in Guadeloupe in 2003–2004. We showed a high seroprevalence in equines in 2003 related to biome, followed by a major decrease in virus circulation in 2004. No human or equine cases were reported during the study.

The recent introduction of West Nile virus (WNV, family Flaviviridae, genus *Flavivirus*) into the Caribbean region is a major public health concern, particularly because transmission of this virus probably occurs year-round in the neotropics. Since 2002, WNV activity has been detected in Guadeloupe (1), Mexico (2,3), the Dominican Republic (4), and Jamaica (5). The objectives of this study were to determine the spatial distribution of WNV in Guadeloupe, establish a surveillance system in humans and animals to detect clinical cases, and increase our understanding of WNV epidemiology in the neotropics.

## The Study

The investigation was conducted in the Guadeloupe archipelago, which includes Guadeloupe (the main island), Marie Galante, Saint Martin, and Saint Barthelemy. Surveys of domestic birds (chickens) were performed in July 2003 and 2004 on 25 to 27 farms selected to cover the whole island; each farm contained 15–20,000 chickens from 1 month to 2 years of age. Exhaustive surveys were also conducted on equines in July 2003 and August 2004 (46 survey locations, 1–68 equines each, mean 10 equines).

Epitope-blocking enzyme-linked immunosorbent assays (ELISA) were performed by using the WNV-specific monoclonal antibody 3.1112G and flavivirus-specific monoclonal antibody 6B6C-1 (Chemicon, Temecula, CA, USA) as previously described (6,7). The ability of the test sera to block the binding of the monoclonal antibodies to WNV antigen was compared to the blocking ability of horse or chicken serum without antibody to WNV. An inhibition value >30% was considered to indicate the presence of viral antibodies. Plaque reduction neutralization tests (PRNTs) were performed as described previously (3) on serum samples that had antibodies to flaviviruses. PRNTs were performed with WNV and St. Louis encephalitis virus (SLEV, family Flaviviridae, genus *Flavivirus*). A serum sample was considered to have antibodies to WNV if it significantly inhibited the binding of monoclonal antibody 3.1112G by blocking enzyme-linked immunosorbent assay (ELISA) and had a 90% plaque reduction (PRNT_90_) titer to WNV that was at least 4-fold greater than the corresponding SLEV PRNT_90_ titer. A serum sample was considered to have antibodies to SLEV if it inhibited the binding of monoclonal antibody 6B6C-1 and had a PRNT_90_ titer to SLEV that was at least 4-fold greater than the corresponding WNV titer. A serum sample was considered to have antibodies to a flavivirus of undetermined origin if it contained epitope-blocking ELISA or neutralizing antibodies but did not meet the criteria for a WNV or SLEV infection.

Out of 487 equines (437 horses, 34 donkeys, 16 ponies) sampled in July 2003, 94 (19.3%) had antibodies to WNV, and 10 (2.1%) had antibodies to a flavivirus of undetermined origin (Table 1). In August 2004, of 431 equines (386 horses, 27 donkeys, 18 ponies), 70 (16.2%) had antibodies to WNV, and 11 (2.6%) had antibodies to a flavivirus of undetermined origin. WNV PRNT_90_ titers were between 1:20 and 1:1,280 (mean 1:260).

**Table 1:** West Nile virus (WNV) seroprevalence in equines and chickens in Guadeloupe, 2002–2004

Sample	Equines, n (%)			Chickens, n (%)		
	July 2002*	July 2003	August 2004	December 2002*	July 2003	July 2004
WNV positive	10 (2.8)	94 (19.3)	70 (16.2)	11 (52.4)	11 (1.7)	5 (0.6)
Unknown flavivirus positive	0	10 (2.1)	11 (2.6)	0	1 (0.2)	2 (0.2)
Total tested	360	487	431	21	656	801

*Data from the serosurveys conducted in 2002 have been presented elsewhere (1).

**Figure 1:**
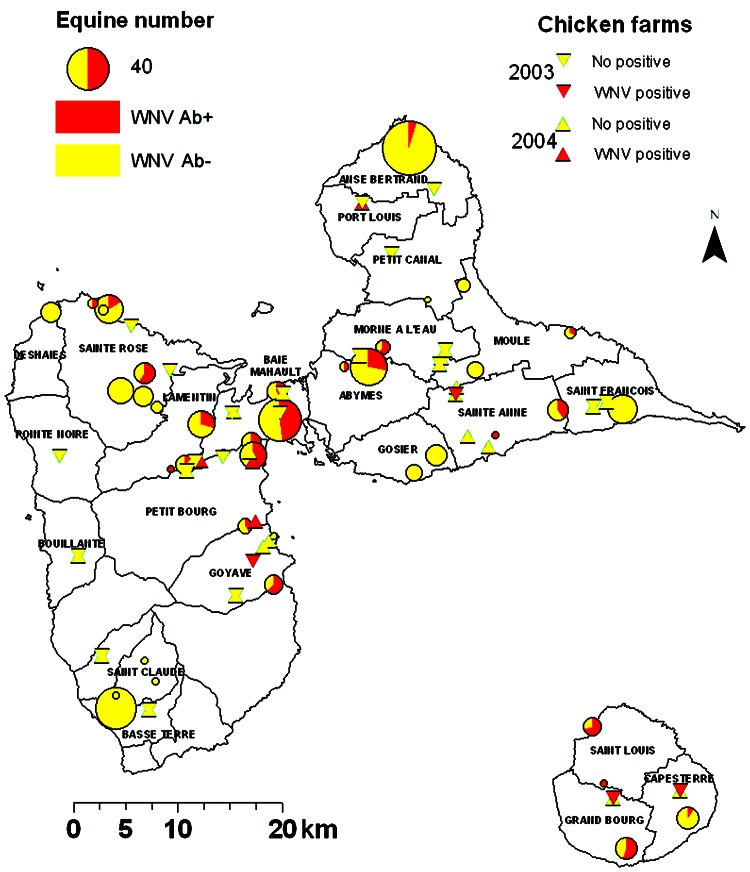
Results of West Nile virus (WNV) serosurveys in chickens and equines in Guadeloupe, 2003–2004. Equine centers are represented by circles, the sizes of which are proportional to the numbers of equines. The proportion of WNV-seropositive animals is represented in red. Chicken farms are represented by triangles (pointing down for 2003 survey, pointing up for 2004 survey). Red triangles denote farms where at least 1 seropositive chicken was identified. All other farms are denoted by yellow triangles. Ab, antibody.

In 2003, WNV seroprevalence in the 46 equine centers was highly heterogeneous (0%–100%, chi-square test p<0.001, Figure 1). This heterogeneity was also found by county (0%–71%, p<0.001) and by island (p<0.001, Table 2).

**Table 2:** West Nile virus (WNV) seroprevalence in equines by island, July 2003

Island	WNV antibody positive, n (%)	No. tested
Guadeloupe main island	80 (19.6)	409
Marie Galante	13 (43.3)	30
Saint Martin	1 (2.9)	34
Saint Barthelemy	0	14

**Figure 2:**
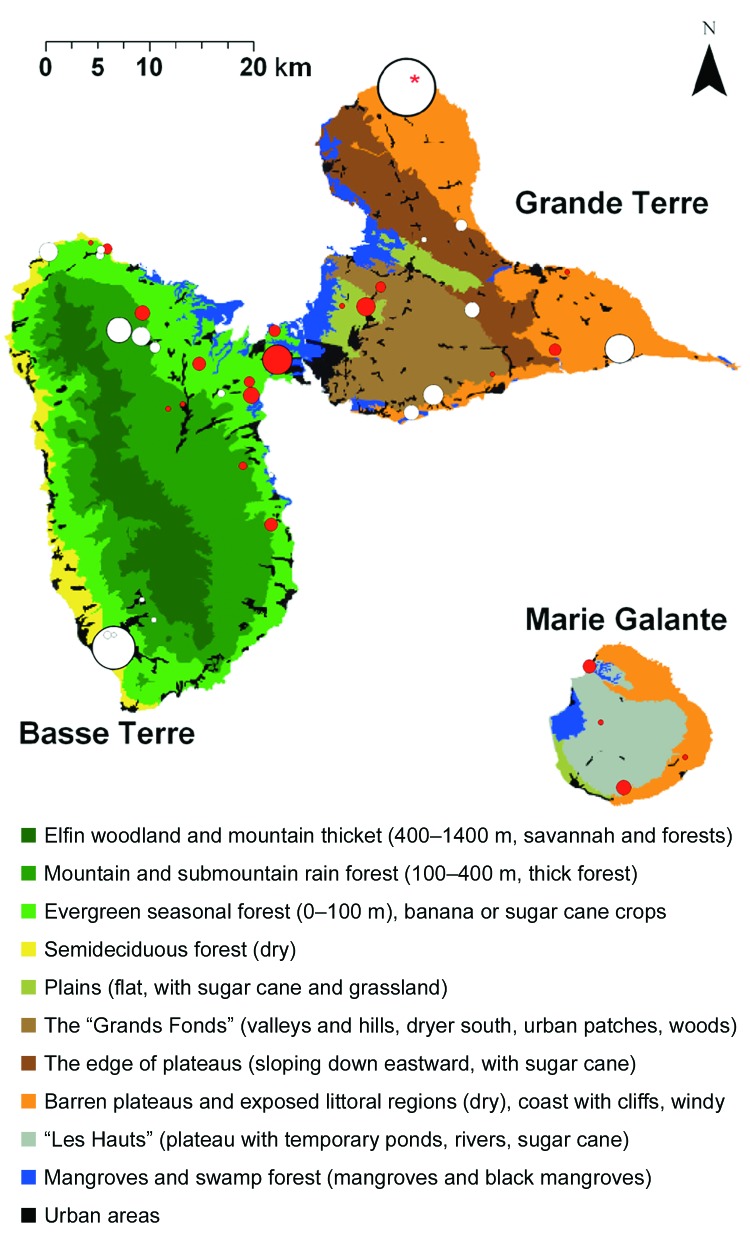
Ecologic map of Guadeloupe and West Nile virus (WNV)-positive equine centers. Basse Terre (southwest) is mainly mountainous (volcanic, highest point 1,467 m) and wet. Grande Terre (northeast) is flat (mainly <100 m) and dry. Marie Galante is flat (plateaus <200 m) but has more water than Grande Terre. The ecologic map was derived from "Carte écologique de la Guadeloupe," created by Alain Rousteau, University Antilles-Guyane. Equine centers with WNV-seropositive equines are represented by red circles (the size of each circle is proportional to the number of seropositive equines). Centers without WNV-seropositive equines are represented by white circles (the size of each circle is proportional to the number of equines tested). The red asterisk shows a site that contained 3 seropositive equines. All were race horses that travel frequently and thus may have been infected elsewhere.

Figure 2 shows each equine center in relation to the ecologic area. Most WNV seropositive equines were located in evergreen forests characterized by a low altitude (≤100 m) and intensive farming of sugar cane in the vicinity of mangroves (*Rhizophora mangle*, *Avicennia germinans*, *Laguncularia racemosa*, *Conocarpus erectus*), back mangroves (marshy forests with *Pterocarpus* spp.), and swamps. Within Grande Terre, seropositive equines were mostly identified in the humid plains of Abymes or near small mangrove areas. In Marie Galante, nearly all seropositive equines were located near swamps, temporary ponds, or rivers. Conversely, no seropositive equines were found in the semideciduous forest (dry leeward coast of Basse Terre) and very few in the dry regions of Grande Terre.

Sixty-two equines that were WNV seronegative in December 2002 (1) were retested in July 2003, and all remained seronegative. Because of changes in the whole population (e.g., death, exportation), only 328 equines were tested in both July 2003 and August 2004. Of the 257 seronegative equines identified in 2003, 256 were seronegative in 2004, and 1 had been infected with an undetermined flavivirus. Thus, no WNV seroconversions were observed in equines between 2003 and 2004. Conversely, all the WNV-seropositive equines in 2003 were still positive in 2004.

In July 2003, of 656 chickens, 11 (1.7%) had antibodies to WNV, and 1 had antibodies to an undetermined flavivirus. Three (0.5%) WNV-seropositive chickens were found on the main island of Guadeloupe and 8 (14.8%) on Marie Galante (Figure 1). In July 2004, of 801 chickens, 5 (0.6%) had antibodies to WNV, and 2 had antibodies to an undetermined flavivirus. All seropositive chickens were on the main island of Guadeloupe (Figure 1). WNV PRNT_90_ titers were between 1:20 and 1:1,280 (mean 1:519).

A surveillance system was established in equines, domestic and wild birds, and humans. Equine surveillance was performed by veterinarians. Awareness of WNV was increased in the public, hunters, and horse and poultry owners with educational leaflets and with the help of veterinarians and natural reserve wardens. In 2003 and 2004, veterinarians reported 4 horses that exhibited signs of WNV-like illness (ataxia, weakness of limbs); the horses were tested at the onset of clinical signs and 15 days later. All were negative for WNV by epitope-blocking ELISA and PRNT, and other causes were eventually determined. Clinical signs were not observed in any equines with antibodies to WNV. Abnormal death and paralysis were reported in domestic chickens in 5 farms. In these farms, serum specimens from 23 chickens were analyzed for evidence of WNV infection, and 1 chicken was seropositive.

Human surveillance was performed by the public hospitals of Guadeloupe. In humans, suspected cases were defined as neurologic symptoms of encephalitis or meningoencephalitis or acute neurologic symptoms with fever. In 2003, the hospital reported 9 suspected cases of clinical WNV infection in Guadeloupe (Cecile Herrmann-Storck, pers. comm.). Antibodies to WNV were not detected in the sera of any of these patients, and another cause was determined.

## Conclusions

A high seroprevalence (19.3%) for WNV was detected in equines in 2003. Seropositive equines and chickens were commonly found near mangroves, which contain many species of wild birds and mosquitoes. *Culex nigripalpus* and *Ochlerotatus taeniorhynchus* are common in Guadeloupe and are particularly abundant in mangrove areas. *Culex* species are the major amplification vectors of WNV in the United States (8,9) and may also be vectors of WNV in Guadeloupe.

Results of the equine and avian serosurveys suggest that transmission of WNV decreased dramatically in 2003 and 2004 in comparison to 2002. No equine seroconversion occurred between January 2003 and August 2004, and seroprevalence in chickens was low in 2003 (1.7%) and 2004 (0.6%). In comparison, 10 of 21 chickens in Goyave were seropositive for WNV in 2002, although the sample size was small (1). In the tropics, where temperature is favorable year-round, changes in rainfall can substantially affect the size of vector populations (10). *Cx. nigripalpus* and *Oc. taeniorhynchus* need heavy rains or changes in water level to develop (11). Therefore, the 7-month drought (half of the usual rainfall) observed between November 2002 and May 2003 was probably responsible for a major decrease in the mosquito population. If *Cx. nigripalpus* is involved in WNV transmission in Guadeloupe, a decrease in its population could explain reduced virus circulation. Alternatively, the number of nonimmune resident birds may have decreased.

No dead wild bird was reported to the veterinary services in Guadeloupe in 2003 or 2004, although dead bird carcasses are presumably difficult to find in areas with dense vegetation. No abnormal death rate was noted in anthropophilic birds either.

Although an efficient passive surveillance system was implemented in humans and equines, no clinical cases of WNV infection were observed. This situation is considerably different from that observed in the United States but mimics the situation in Mexico, where only a few human and equine cases were observed, despite a seroprevalence of 29% in equines in 2004 (12). Cross-protection conferred by other flaviviruses could explain the difference with the North American situation. In fact, we found some equines that were considered to have been infected with an unknown flavivirus. Alternatively, virus mutations could explain a change in virulence and the absence of clinical cases. Indeed, recent studies identified attenuated WNV in Texas (13) and southern Mexico (14). Isolation and characterization of WNV in Guadeloupe would help clarify these issues.
